# The Devil Is in the Tail: Manchester Short Assessment of Quality of Life (MANSA) test percentiles and normalized T-scores

**DOI:** 10.1007/s10862-025-10266-0

**Published:** 2026-01-28

**Authors:** Edwin de Beurs, Bertus F. Jeronimus

**Affiliations:** 1 Department of Clinical Psychology, Leiden University, Netherlands & Arkin Mental Health Institute, Amsterdam, Netherlands; 2 Department of Psychology, University of Groningen, Groningen, Netherlands

**Keywords:** T-scores, Percentile rank scores, MANSA, Substance Use Disorder

## Abstract

**Purpose:**

In measurement-based care or routine outcome monitoring self-report questionnaires are used to monitor how a patient fares in therapy. Interpretation and utilization of test results are improved and communication facilitated when raw scores are converted to common metrics, such as standard scores (T-scores) and percentile rank scores. Both types of common metrics are described and reviewed and a warning is issued that percentiles may be misinterpreted at the tails of the scale.

**Methods:**

Data from the Manchester Short Assessment (MANSA) was used to investigate various approaches to obtain T-scores. The study analyzed cross-sectional data from two normative samples: a representative sample of the Dutch general population (N = 11,789) and a clinical sample (N = 9987) of patients with substance use disorder. Linear, normalized, and IRT-based T-scores were compared.

Results revealed that T-scores derived from a linear conversion were biased at the lower end of the scale. Normalizing raw test scores through either Rankit normalization or by an IRT approach yielded improved and quite similar T-scores. For all possible raw scores on the MANSA, corresponding normalized T-scores are presented, as well as Percentile Rank scores for the two reference groups. Finally, cut-off values for reliable change and clinically significant change are presented for raw scores and T-scores.

**Conclusion:**

Practical guidance is offered for converting raw test scores into two common metrics: normalized T-scores and Percentile Rank scores. For T-scores, simple linear conversions yielded biased results. These findings have implications for test developers, practitioners, and researchers who want to express test results in valid and unbiased common metrics.

**Supplementary Information:**

The online version contains supplementary material available at https://doi.org/10.1007/s10862-025-10266-0.

## Introduction

The goal of health care and preventive medicine is to improve health and quality of life (Kaplan & Hays, 2022). To express, compare and interpret test scores across different contexts, populations, and time points, requires a common metric (de Beurs et al., 2022). Common metrics can help to correctly interpret test results and enhances the utility and practicality of testing for various purposes, such as educational assessment, clinical diagnosis, and treatment evaluation. Educational researchers typically express test results in common metrics, such as IQ-scores (*M* = 100; *SD* = 15), and T-scores (*M* = 50, *SD* = 10), as a special cases of a standardized score or Z-score. However, clinical psychology is lagging behind with the application of common metrics, which is unfortunate as it hampers the implementation of measurement based care (Fortney et al., 2017). However, there appears to be renewed interest in the use of common metrics (Reynolds et al., 2021), such as percentiles (Crawford & Garthwaite, 2009) and T-scores, for instance in the Patient-Reported Outcomes Measurement Information System (PROMIS) initiative (Rothrock et al., 2020). In the present study, we used data collected with the Manchester Short Assessment of Quality of Life (MANSA; (Priebe et al., 1999) in two sizable samples: a sample from the Dutch general population and a clinical sample of patients treated for Substance Abuse Disorders (SUDS). The MANSA is one of the most used instruments in psychiatry to examine well-being in clinical populations, such as physical, cognitive, and sexual function. Recovery in terms of improvement of the MANSA score is deemed by some as more important than symptom reduction (Kilbourne et al., 2018). Routine Outcome Monitoring (ROM) by the patient in mental health care practice relies on an appropriate interpretation of standardized self-reported health and quality of life assessments, such as the MANSA (Kaplan & Hays, 2022; Nugter & Teer, 2011). We aim to present psychometric properties, common metrics, norms, and appropriate cut-off values for reliable change and recovery of the MANSA. Below we first review two common metrics and briefly discuss the choice of appropriate reference groups.

### Percentiles vs. Standard Scores (T-scores)

Percentiles rank (PR) scores express a raw score as the percentage of respondents of a reference group who obtained comparable or lower scores. Typically, half of the respondents whom obtained exactly the raw score are added to the number of respondents with a lower score to calculate the PR-score. An advantage of PR-scores is their simplicity and ease of interpretation, and their ability to capture the relative standing of test takers within a reference group (Crawford & Garthwaite, 2009). Disadvantageous of the PR-score is that it yields an ordered variable with non-equidistant scale points: differences between scores are exaggerated around the mean, and downplayed at the extreme low and high end of the scale, because the bell-shaped normal distribution has most data concentrated near the center with data tapering off towards the extremes. Consequently, differences between PR-score may be misinterpreted at the poles of the scale (Bowman, 2002).

T-scores are basically Z-scores transformed to a more convenient metric. Z-scores are calculated frow raw test scores (RS) through Z = (RS-M)/SD, have a practical range of −3.00 to 3.00 and a mean of M = 0 (SD = 1). This implies decimals and negative scores. T-scores are calculated as T = 10*Z + 50, have a mean of M = 50, SD = 10 and a practical range between 20 and 80. Provided that the raw scores have a normal distribution, T-scores have an interval scale, thus differences between scores are similar for the entire range of the scale.

### General Population vs. Clinical Reference Group

An appropriate reference group is crucial to interpret test results and clinical psychologists often choose between the general population and a clinical sample. The PROMIS health care initiative has chosen the general population as their reference (Kaplan & Hays, 2022); the mean level of measured constructs is typically stable in the general population, whereas means among diverse clinical studies and conditions (e.g., outpatients and inpatients) diverge, which complicates the selection of an appropriate comparison group. Therefore, expressing the severity of a health problem in terms of its difference from the general population mean seems to be an obvious choice. IQ scores also describe intellectual capacity in references to the general population mean, and not relative to intellectually disabled or gifted people. However, compared to the general population as a reference group, clinical norms can sometimes provide more valuable information, particularly regarding PR scores. Using the general population as a reference group for PR scores, can lead to a problem of a restricted range in scores. Clinical subjects will predominantly score in the high range of the percentile scale (in the upper 20%), and thus the range of PR scores will be restricted to 80–100, a small range to express differences in severity among patients, or to express change over time. In contrast, by using a clinical reference group of patients treated at the clinic, the clinician will be able to utilize the full spectrum of the PR scale (0–100) when assessing patient severity and treatment outcome. While this is also true to some extent for T-scores, they are less prone to a limited range, because T-scores are designed to have equal intervals. Most patients will score between T = 55 and T = 80 at the onset of treatment and around 50–55 when treatment was successful. However, selecting the general population as a reference group can introduce the problem of non-standard normal distributions of scores.

### Non-normal Raw Score Distributions

When clinical measures, such as the Brief Symptom Inventory (Derogatis, 1975) or the Beck Depression Inventory (Beck & Steer, 1987), are administered in the general population, the frequency distribution of RS usually does not follow the normal or Gaussian curve. RS tend to be negatively skewed with a tail to the right and peaked due to many respondents scoring in the lower range of the scale, or having the lowest possible score, also called zero-inflation (Magnus & Liu, 2018). RS of measures for functioning or quality of life (QoL) tend to have positive skew due to overrepresentation of high scores in the general population. If RS are not normally distributed, a simple linear transformation of RS into T-scores based on *M* and *SD* [T = 10*Z + 50, with Z = (RS-*M*)/*SD*] will yield skewed and distorted T-scores, leading to an inaccurate interpretation of test results. This is due to the fact that the mean and standard deviation are no longer appropriate estimates of centrality and dispersion to describe the frequency distribution of the skewed scores. To fix this, the questionnaire can be redesigned or adapted to yield a normal distribution of raw scores. Alternatively, raw test scores can be normalized using min–max scaling, or median and IQR, double sigmoid, tanh, Yeo-Johnson, Box-Cox, power, and logarithmic transformations (Peterson, 2021), to mention a few options. Another efficient approach is percentile-based or Rankit normalization of test scores based on the distribution of scores in a reference group (Solomon & Sawilowsky, 2009).

### Classical Test Theory and Item Response Theory

With the advent of Item Response Theory (IRT), two approaches to establishing test scores have become available. According to the classical test theory (CTT) approach RS are usually obtained by simply summing or averaging responses on items of a questionnaire. In contrast, with Item Response Theory (IRT; Embretson & Reise, 2013) the test score is established based on both responses and on the “item response characteristics” of the items that compose a scale. Thus, IRT considers both the item difficulty or severity level parameters and the item score, resulting in a standardized scale score (θ) with a mean of *M* = 0; *SD* = 1. As these θ-scores have a normal distribution, they can be converted to T-scores with T = 10*θ + 50. A requirement for IRT is unidimensionality of the scale, which is usually assessed with confirmatory factor analysis. Also, multidimensional IRT (MIRT; Liu et al., 2018) exists for instruments designed to measure multiple related constructs, but then still unidimensionality of each of the multiple dimensions is required to obtain appropriate factor scores. Scoring according to IRT is more complicated, as it requires an IRT model with item parameters and an algorithm to estimate the factor score.

This ultra-short review of CTT and IRT for the establishment of T-scores indicates that there are two options to obtain normalized T-scores when RS do not have a normal distribution: IRT and normalization with a transformation of RS. Usually, factor scores derived from the IRT approach are normally distributed and a linear transformation will suffice to obtain appropriate T-scores. Normalization of summed or averaged items scores according to CTT may require a mathematical transformation before these are converted to normalized T-scores. If such normalization is required, this will result in a non-linear conversion of RS to T-scores.

### Aim of the Present Research

The primary aim of this research was to investigate the degree of bias when using simple linear transformations to establish T-scores. A secondary aim was to compare two approaches or normalization of RS: Rankit normalization and Item Response Theory (IRT)-based score transformation. We sought to determine the appropriateness of separate norms for different gender and age groups by investigating score differences across these demographics. Finally, we established cut-off values for statistically reliable change and clinical significance, as defined by Jacobson et al. (RCI and CS; Jacobson & Truax, 1991; Jacobson et al., 1999). These cut-off values can be used to determine whether a patient's change in score exceeded measurement error and whether a patient's most recent score falls within the range of the general population.

We first established whether the MANSA meets requirements for IRT (unidimensionality) and whether RS of the MANSA had a normal distribution. RS were expected to deviate from normality. A percentile-based transformation (Rankit) was applied and a non-linear transformation established. To assess the extent of bias, we compared linear-based T-scores with percentile-based Rankit derived normalized T-scores and IRT-based normalized T-scores. We also present and compare PR-scores for both samples.

## Method

### Participants

Data were used from the general population sample of a crowd-sourcing study into the mental health of the general Dutch population “HowNutsAreTheDutch” (van der Krieke et al., 2016) which comprised 12 503 respondents of whom 11 789 completed the MANSA (among other questionnaires; see for a full description of the study Van der Krieke et al. (van der Krieke et al., 2016). Representativeness of the sample for the Dutch general population was investigated: The proportion of urban and rural respondents concurred adequately with the population density in the Netherlands. Women and older respondents were overrepresented and, regarding educational background, the lower educated were underrepresented in the sample (See for more details supplements of Van der Krieke et al. (van der Krieke et al., 2015).

A clinical sample was composed of patients seeking treatment for Substance Use Disorder at the Jellinek clinic (n = 9 983; a convenience sample of consecutive patients). To protect privacy of the patients, clinical data were anonymized and only information about gender and age was preserved.

The population sample contained 3 858 men (32.7%) and 7 931 women (67.3%), mean age was 44.64 (SD = 14.62) years; the clinical sample contained 7 326 men (73.4%) and 2 657 women (26.6%), mean age was 40.41 (SD = 13.19) years. Thus, women were overrepresented in the general population sample and underrepresented in the clinical sample. The clinical sample was on average 4 years younger compared to the population sample.

To investigate the effect of gender and age on the MANSA scores, we compared the scores of respondents who had identified themselves as men and women for both samples. The effect of age was established by dividing both samples in 10 age brackets of roughly equal size: 18–24 years, n = 2 362; 25–29 years, n = 2 706, 30–34, n = 2 364, 35–39 years, n = 2 179, 40–44, n = 2 161, 45–49 years, n = 2 363, 50–54, n = 2 380, 55–59 years, n = 2 249, 60–64, n = 1 631, 65 + years, n = 1 377.

### Procedure

Data from the general population were collected through a crowd sourcing procedure, comprehensibly described by van der Krieke et al. (2016) as part of a larger study investigating the mental health of the adult (18 +) Dutch general population though momentary assessment and survey research (HowNutsAreTheDutch). The size of the normative group was N = 11 789.

For the clinical sample, data were used from a convenience sample of N = 9 983 patients with Substance Use Disorder, seeking treatment at the Jellinek clinic, specialized in SUD treatment for in- and outpatients. The Jellinek is a clinic of Arkin, the largest mental health care provider of Amsterdam, the Netherlands. Data were collected prior to treatment as part of Routine Outcome Monitoring (de Beurs et al., 2011) and were anonymized before use. Due to anonymization of the patient data, only age and gender were known. In the general population sample 32.7% were males, mean age was *M* = 42.70 (*SD* = 14.62); in the clinical sample 73.4% were males, mean age was *M* = 44.64 (*SD* = 14.62)..MANSA.

The Manchester Short Assessment of quality of life (MANSA; Priebe et al., 1999) is a brief questionnaire designed to assess overall quality of life (QoL) in patients with severe mental health problems. It can be completed by a clinician (as a rating scale or in interview form) and it can be used as a self-report measure. It was developed to provide a simple and efficient tool for clinicians to assess objective aspects and subjective satisfaction with life of a patient. Part of the MANSA consists of 12 “subjective” items, which are rated on a 7-point Likert scale (0 = Couldn’t be worse, Displeased, Mostly dissatisfied, Mixed, Mostly satisfied, Pleased, 7 = Couldn’t be better). These 12 items cover a range of QoL domains including social relationships, work and leisure activities, finances, safety, living conditions, sex life, and mental and physical health. An additional four items are more objective questions (Do you have anyone who you would call a “close friend”?) with a Yes/No answer format. These items were excluded from the present analysis. The scores for the remaining 12 items (7-point scale) are summed to produce a total score, which represents the overall satisfaction with QoL. Higher scores indicate higher satisfaction. Support has been found for good psychometric properties of the MANSA, such as a sufficient internal consistency reliability: Cronbach’s alpha is α =.74 (Priebe et al., 1999) to α =.81 (Björkman & Svensson, 2005), and both studies report medium to high correlations with other measures of QoL, supporting convergent validity. Although one study reported a 2-factor structure for the 12 subjective items of the MANSA (Petkari et al., 2020), generally a total score is established which ranges from 12 to 84 (Priebe et al., 1999; van Nieuwenhuizen et al., 2024).

The study used the Dutch translation of the MANSA by van Nieuwenhuizen (2017). The good psychometric properties were preserved in the translation, such as internal consistency (Cronbach’s α =.84 for the sum score of the 12 items and satisfactory convergent and divergent validity indices Priebe et al., 1999; van Nieuwenhuizen et al., 2024).

### Statistical Analysis

All statistical analyses were done with R (R Core Team, 2020), version 4.4.1. Psychometric properties (*M, SD,* Skewness, and Kurtosis) were established for the raw scale scores from the general population sample. Next, the IRT requirement of unidimensionality of the MANSA was checked with the package lavaan (version 06.12; Rosseel, 2012). Items were considered ordered, analyses were based on the polychoric correlation matrix, and robust indicators of fit were evaluated as suggested by Hu and Bentler (1999): RMSEA <.08, SRMR < 0.08; CFI > 0.95; TLI >.095.

The multidimensional IRT (mirt) package of R, version 1.37.1 (Chalmers, 2012) was used to determine relevant item characteristics and to build an IRT-model for the MANSA in order to obtain scale scores (factor score or θ’s) from item responses, using the “Graded Response Model for polytomous items” with the Expected-A-Posteriori score (EAP) as estimator. The IRT model was fitted with multiple group estimation with the general population or clinical sample as the grouping factor, and item parameters were fixed to be equal across groups. The latent trait (θ) was standardized to a scale with a mean of 0 and a standard deviation of 1 for the general population in order to obtain Z-scores, a higher θ meaning more satisfaction with QoL. The IRT factor scores were multiplied by 10 and 50 added to obtain a θ-based T-score for each subject.

**Fig. 1:**
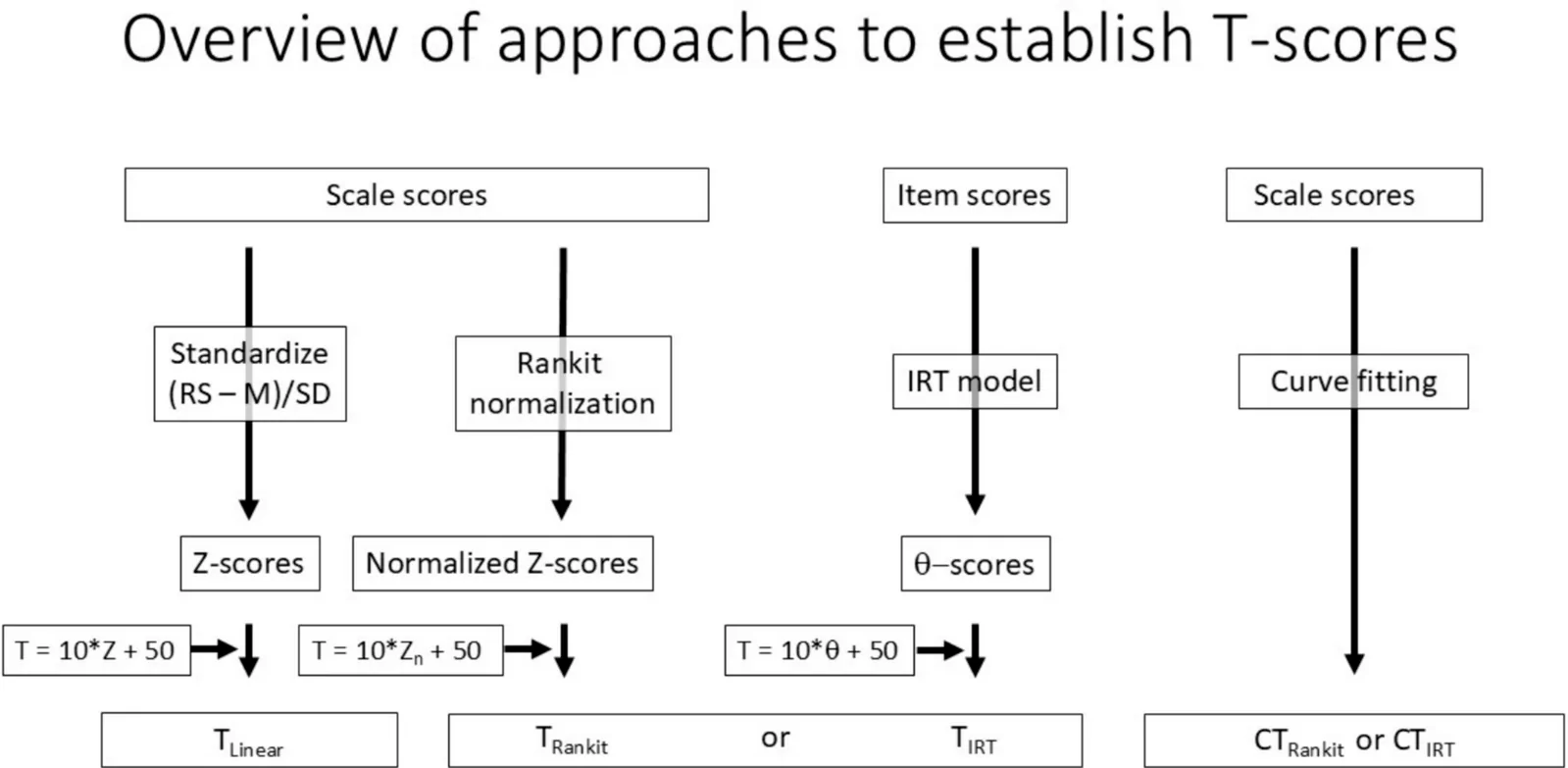
Overview of approaches to establish T-scores. Note: RS = raw test result; M = mean of the reference population, SD = standard deviation; θ = theta or factor score derived from IRT model; T_Linear_ = T-score resulting from a simple linear transformation; T_Rankit_ = Rankit-based T-score; T_IRT_ = θ-based T-score; CT_Rankit_ = T-score as calculated with a curvi-linear rankit-based function; CT_IRT_ = T-score as calculated with a curvi-linear IRT-based function

Linear T-scores were calculated by multiplying standardized scores by 10 and adding 50 using the general population sample as reference group. These T_Linear_-scores were compared to T_IRT_ and to T_Rankit_-scores, that were based on normalized raw scores through the Rankit approach (Solomon & Sawilowsky, 2009) with the RankNorm function in the R RNOmni package version 1.0.1 (McCaw, 2019). Figure 1 present an overview of the various approaches for establishing T-scores. Finally, PR scores were obtained with the RankNorm function as well. Two PR scores were established: PR_n for the general population sample and PR_cl for the clinical sample.

We established a cross-walk table to convert raw scores to the common metrics: T-scores and PR-scores. In this table, T-scores were based on θ’s from the IRT model (T_IRT_). Finally, formulas were established to convert raw scores into normalized T-scores and PR-scores computationally. These formulas were found through non-linear modelling (the Non-linear Least Squares nls function in the R Stats (version 3.6.2) package and the glsnls package version 1.2.0 (https://cran.r-project.org/web/packages/gslnls/gslnls.pdf). These formulas can be built into an Excel file or in questionnaire scoring software.

## Results

### Frequency Distribution of MANSA Scores

Note under Table 1: *M* = mean; *SD* = standard deviation; Skew. = Skewness,; Kurt. = Kurtosis; *W* = the result of a Shapiro-Wilks test for normality of the data (shapiro.test function in R) (Hernandez, 2021; Shapiro & Wilk, 1965).

**Table 1:** Mean, SD, Skewness, and Kurtosis of items and the summed score of the MANSA in the general population sample

	M	SD	Skew	Kurt	W	M	SD	Skew	Kurt	W
Item 1	4.91	1.12	−0.65	0.46	0.89	3.91	1.70	−0.06	−1.09	0.93
Item 2	5.41	1.21	−1.17	1.41	0.84	4.81	1.98	−0.66	−0.89	0.86
Item 3	5.10	1.34	−1.03	0.53	0.86	4.81	1.87	−0.59	−0.85	0.88
Item 6	5.09	1.26	−0.86	0.57	0.88	3.90	1.81	−0.04	−1.22	0.92
Item 7	5.02	1.09	−0.71	0.52	0.89	3.63	1.80	0.13	−1.23	0.91
Item 8	5.57	1.10	−1.21	1.76	0.83	3.38	1.79	0.31	−1.14	0.90
Item 11	5.94	0.74	−1.54	6.09	0.73	5.53	1.44	−1.32	1.17	0.80
Item 12	5.46	1.21	−0.83	0.62	0.89	4.41	1.76	−0.35	−1.04	0.91
Item 13	4.39	1.56	−0.45	−0.59	0.92	4.65	1.80	−0.55	−0.84	0.90
Item 14	4.98	1.22	−0.76	0.49	0.92	4.43	1.96	−0.32	−1.17	0.90
Item 15	4.99	1.22	−0.80	0.30	0.88	4.00	1.91	−0.09	−1.26	0.91
Item 16	4.86	1.37	−0.70	−0.10	0.89	3.71	2.02	0.04	−1.43	0.87
Total	61.72	8.76	−0.81	0.96	0.96	51.14	14.20	−0.17	−0.57	0.99

Note: M = mean; SD = standard deviation; Skew. = Skewness,; Kurt. = Kurtosis; W = the result of a Shapiro-Wilks test for normality of the data (shapiro.test function in R) (Hernandez, 2021; Shapiro & Wilk, 1965)

The distribution of MANSA scores for both the population-based sample and the clinical sample in Table 1 shows key statistics, such as the mean, standard deviation (SD), kurtosis, and skewness, and the W test statistic for normality. For the population-based sample mean scores on the MANSA items were generally high and negatively skewed due to an underrepresentation of low scores. The MANSA total score approached a normal distribution (skewness and kurtosis < 1.00), but was still flagged by the W statistic as deviating significantly from normality. MANSA scores from the clinical sample showed less skew, with the exception of item 11 (satisfaction about personal safety), due to an overrepresentation of high scores. Table 1 also present results of t-test comparing scores from both groups. Scores differed substantially between both groups, amounting to Cohen’s *d* = 1.3–2.8, underlining the known-group validity of the MANSA.

The population sample reported substantially higher mean QoL (*Mean* = 61.72, *SD* = 8.76) than the clinical sample (*M* = 51.14, *SD* = 14.20); Cohen's *d* = 0.91 ([0.89, 0.94]; two-sided *t*-test *t*_(16036.92)_ = 64.76, *p* <.001). In the population sample, women reported slightly lower mean QoL than men, but the magnitude of this effect was negligible (Cohen's *d* = 0.04 [0.00, 0.08]; 7931 women, *M* = 61.60 [*SD* = 8.49] versus 3858 men, *M* = 61.98 [*SD* = 9.27]; two-sided *t*_(7074.53)_ = 2.15; *p* = 0.03).

In the clinical sample, women also reported lower QoL than men, but again this effect was negligible (*d* = 0.08 [0.03,0.12]; *n* = 2 657 women, *M* = 50.32 [*SD* = 13.54]; *n* = 7 326 men, *M* = 51.43 [*SD* = 14.42]; *t*_(4985.36)_ = 3.58; *p* <.001). Consequently, gender norms proved unnecessary.

Older adults reported significantly higher QoL (Cohen's d = 0.26 [0.24, −0.29]), which rose with a medium size effect from young adults (aged 18–44, *n* = 11 722, *Mean* = 55.34 [*SD* = 13.05]) to older adults (aged ≥ 45, *n* = 10 000, *M* = 58.66 [*SD* = 12.07]; *t*_(21612.81)_ = 19.51; *p* <.001).

**Fig. 2:**
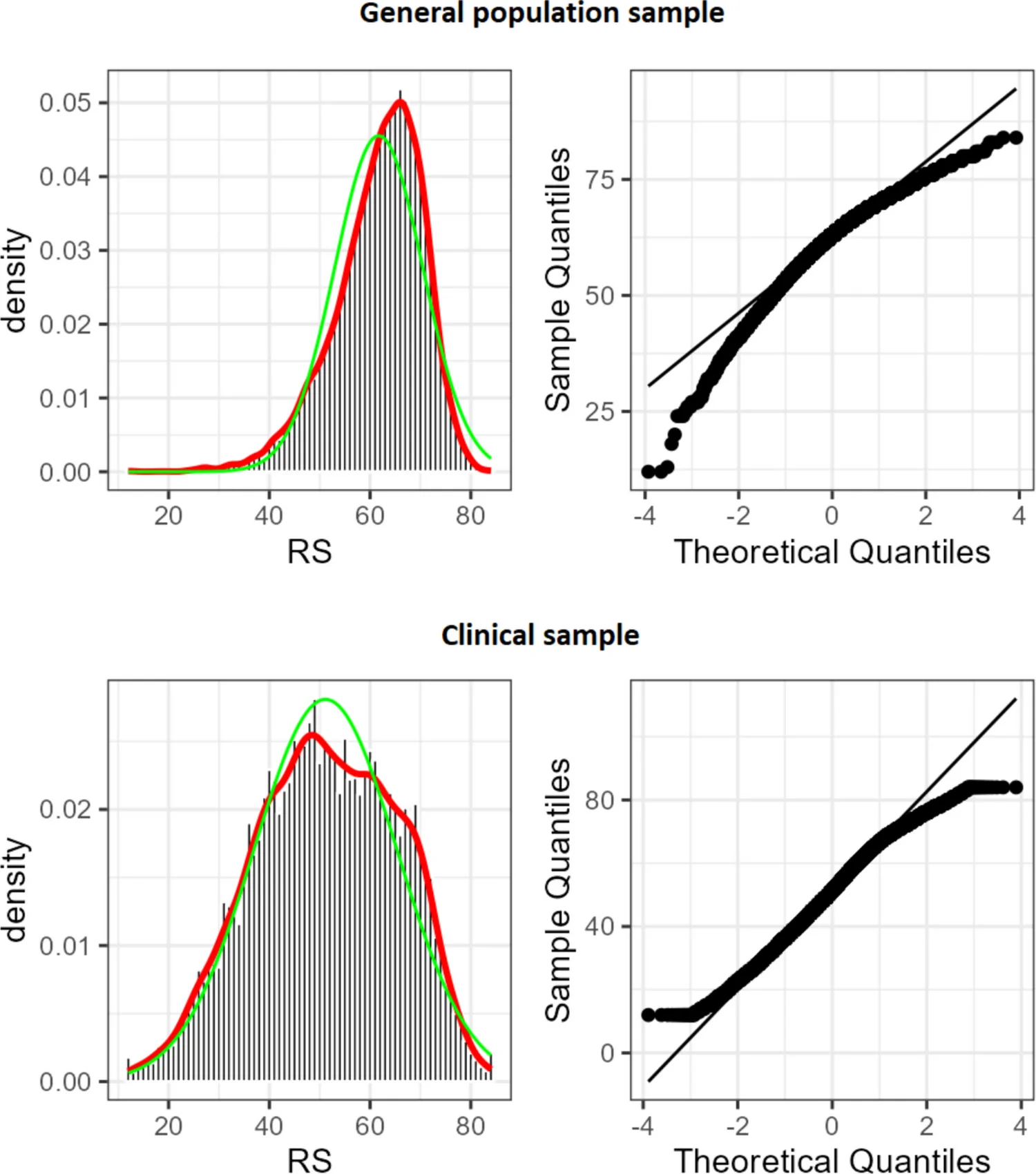
Histogram with a Density Curve (red) and a Normal Curve (green) and p-p Plots for the General Population (Upper Two Graphs) and the Clinical Sample (Lower Two Graphs)

Figure 2 shows density plots with density and normal curves and p-p plots for the general population (upper two graphs) and the clinical sample (lower two graphs), illustrating the non-normal distribution of the MANSA scores in both populations. The general population data are skewed to the left and peaked; the clinical data is somewhat platykurtic.

### IRT Results

First, the unidimensionality of the factor structure of the MANSA was investigated. A parallel analysis revealed a first factor with eigenvalue = 4.49 and a second factor with eigenvalue = 1.08. The unidimensionality of the MANSA was further checked by establishing the fit of a single factor model (χ^2^ (54) = 3325.20; *p* <.001; *CFI* =.98, *TLI* =.97; *SRMR* = 0.05; *RMSEA* = 0.07). These indices indicated adequate fit of a single factor model (with the exception of RMSEA). A two-factor model, as proposed by Petkari et al. (2020), setting items 11–13 apart in a second factor, had a lower model fit (χ^2^ (53) = 2996.54) but identical fit indicators. Hence, we concluded that there was sufficient support for unidimensionality of the MANSA. The internal consistency of the single factor was Cronbach’s *α* =.88 and McDonalds Omega (general) of ɷ =.88 for the total sample (N = 21 772).

Next, T_IRT_, T_Linear_, and T_Rankit_ were established according to the approaches depicted in Fig. 1. T_IRT_-scores stemmed from the factor scores of the IRT model (T_IRT_ = 10 * θ + 50). T_Linear_ resulted from the linear transformation of raw scores to Z-scores to T-scores. T_Rankit_ scores were established with the Rankit procedure (RankNorm in R). Finally, we calculated CT_IRT_ from nonlinear regression of T_IRT_-scores onto raw MANSA scores in order to have estimates for each raw score. The various T-score variants are presented in Table 2 for the first 19 raw scores (12 to 30) on the MANSA.

**Table 2:** Various T-scores for MANSA Raw Scores 12 to 30

RS	θ-based T-scores	CT_IRT_	T_Linear_	T_Rankit_
12	2.3	1.9	−6.8	12.4
13	4.3	3.9	−5.6	14.8
14	5.5	5.7	−4.5	NA
15	7.9	7.4	−3.3	NA
16	8.7	9.0	−2.2	NA
17	9.6	10.5	−1.1	NA
18	11.3	11.9	0.1	15.7
19	13.2	13.2	1.2	NA
20	14.9	14.4	2.4	16.3
21	15.6	15.6	3.5	NA
22	16.7	16.7	4.6	NA
23	17.7	17.7	5.8	NA
24	19.0	18.7	6.9	17.6
25	19.7	19.6	8.1	18.6
26	20.6	20.5	9.2	19.4
27	21.7	21.3	10.4	20.7
28	22.1	22.1	11.5	21.8
29	22.6	22.9	12.6	22.3
30	23.6	23.6	13.8	22.6

Note: RS = raw score; θ-based T-score = 10*IRT-factor score + 50; CT_IRT_ = θ-based T-score regressed onto RS; T_Linear_ = linear transformation of RS to T; T_Rankit_ = Rankit based transformation

We investigated the validity of the T-scores by examining the correspondence of θ-based T_IRT_ (the gold standard) with T_Linear_ and T_Rankit_, respectively, with intraclass correlation coefficients (both in the range of ICC = 0.96 to 0.97), and we inspected Bland–Altman plots for correspondence. Formulas to calculate T-scores for both age groups (based on distinct IRT-models) were also established and included in the supplementary materials, along with an excel file, demonstrating the formulas.

**Fig. 3:**
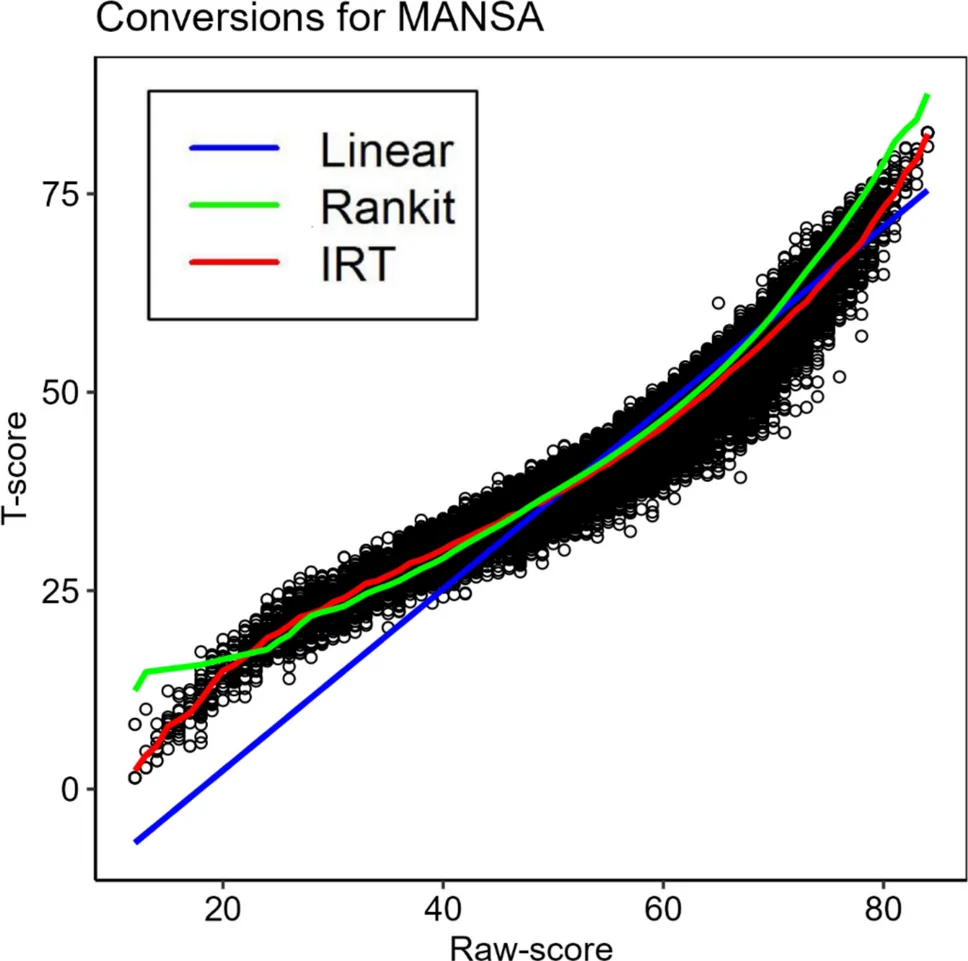
Curves for T_Linear_, T_Rankit_ and T_IRT_ (regressed) Approaches Compared to θ-based T-scores (dots). Note: Dots represent IRT based T-scores; Linear = T_linear, Rankit = curve for Rankit based T-scores, IRT = curve for θ-based T-scores (derived from non-linear regression of T_IRT_ onto RS)

In Fig. 3, the x-axis represents the MANSA raw scores scale (RS) and the y-axis the T-score scale. The figure shows a scatterplot of RS—T_IRT_-score pairs as black circles and curves for the relation between RS and various T-score approximations. T_Linear_ according to the linear relation between RS and T-scores (T = 10*((RS-61.72)/8.76) + 50 = > T = 1.142*RS −20.475) is depicted by the blue line. The green line shows the curvilinear relation between RS and normalized T_Rankit_-scores. The red line shows the curvilinear relation between RS and calculated T_IRT_ scores (obtained by regressing θ-based T scores onto RS with nlm). The vertical dispersion of black circles illustrates that various T_IRT_ are to be found for each RS (RS are summed scale scores, whereas T_IRT_ are based on the IRT model and will vary per RS). The results in Fig. 5 show that the red line for regressed T_IRT_ scores reflected the pattern of RS—T_IRT_ pairs best. However, the green line for T_Rankit_ corresponds closely with the red line for T_IRT_. T_Rankit_ scores were quite similar to T_IRT_, except for the lowest raw MANSA scores (RS = 12–20) where T_Ranit_ scores were too high (a maximum bias of 10 points at RS = 12). Finally, the T_Linear_ scores (the straight blue line) were in the lower- and midrange of the scale (from RS < 50) increasingly lower than the regressed T_IRT_, a bias finally amounting to 15 points at the lowest level.

With ICC we investigated the correspondence between T_linear_ and T_Rankit_ with regressed T_IRT_. We set the limit for a substantial difference at T = 5, as this corresponds to 0.5 *SD* units, which is also frequently chosen as the limit of a minimally important change in score (de Vet et al., 2006). Table 3 presents the results. Figure 3 shows Bland–Altman plots for the correspondence of T_Linear_ and T_Rankit_ with θ-based T_IRT_-scores. The linear conversion yielded depressed T-scores and elevated T scores in the midrange compared to θ-based T_IRT_; there was no evidence for a systematic distortion of T_Rankit_, again using θ-based T_IRT_ as reference point.

**Table 3:** Indicators of Correspondence of T_Linear_ and T_Rankit_ with θ-based T-scores

								< −5		−5 ≤ D ≤ 5		> 5	
scale	ICC	CI95	F	Bias	PercErr	CI95 +	CI95-	n	%	n	%	n	%
T_Linear_	.96	.96—.96	53.81*	−0.06	−5.06	4.94	20.0	575	4.88	11,203	95.03	11	0.09
T_Rankit_	.97	.97—.97	74.39*	−0.06	−4.32	4.20	17.0	180	1.53	11,517	97.69	92	0.78

NB: *p <.001; ICC estimates and their 95% confidence intervals were calculated using R and based on a 2-way mixed-effects model. ICC = Absolute agreement; Bias = difference between both T-scores (negative bias values indicates that θ-based T-scores are higher than T_Linear_ or T_Rankit_); Perc. Err. = Percentage error, the width of the limits of agreement interval divided by the mean T-score of the population [(CI95- + CI95 +)/M]; “D < −5 and D > 5” = the number and percentage of subjects for whom the difference (D) between both T-scores is more than 5 points; “−5 ≤ D ≤ 5” = the number and percentage of subjects for whom the difference between both methods is 5 points or less

**Fig. 4:**
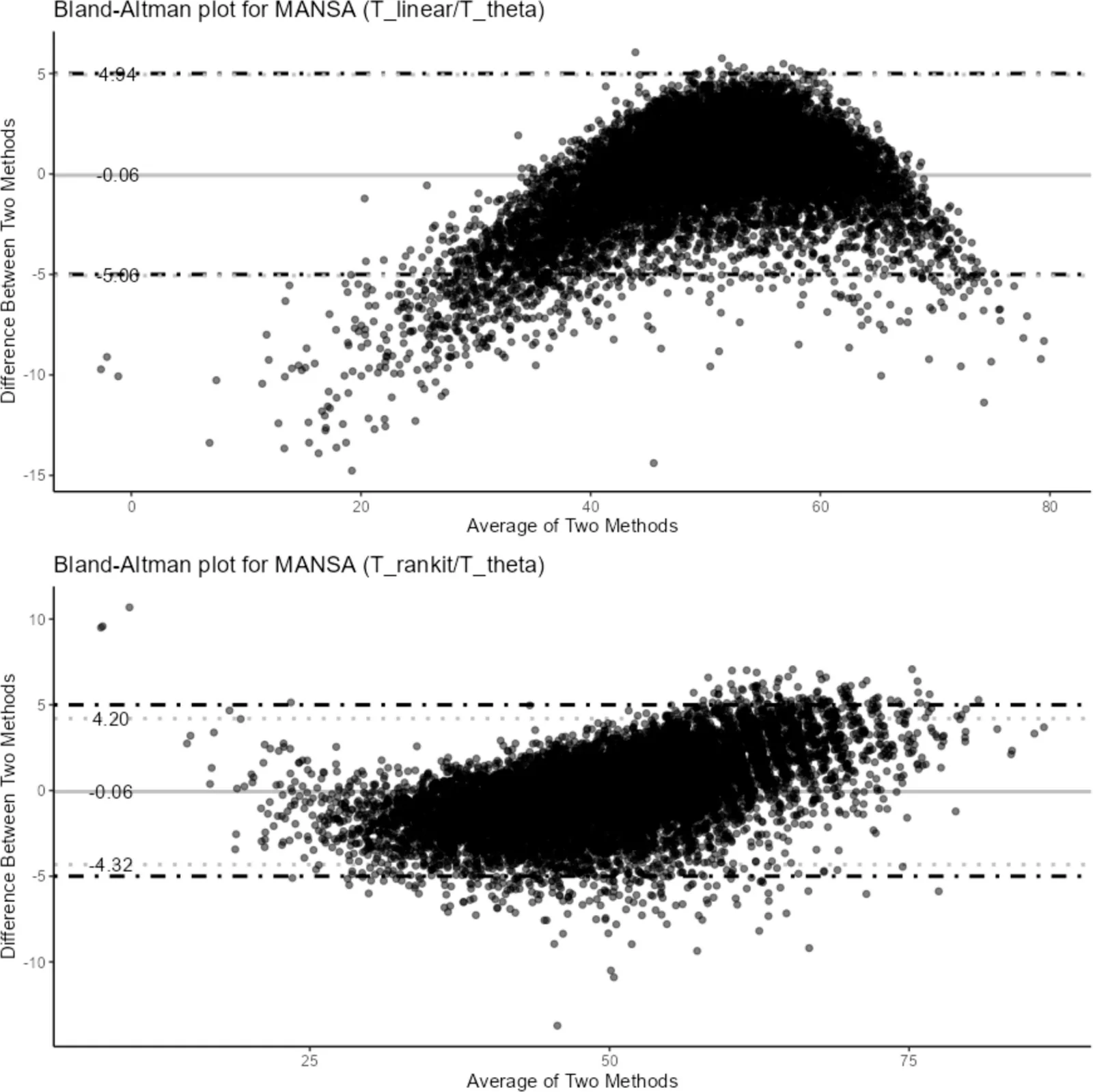
Bland–Altman Plot for Correspondence of T_Linear_ and T_Rankit_ with θ-based T_IRT_-scores. Note: the upper and lower lines indicate the thresholds on agreement (95% of the observation reside between these lines). The line is the middel is the average differences between both aproaches; for T_Linear_ most differences in score pairs are found at the lower end of the scale and are, in some cases, substantial; for T-Rankit, 95% of the score pairs fall within 5 T-score points difference

Figure 4 present two Bland Altman plots for the correspondence between two methods of obtaining T-scores. The upper Bland–Altman plot shows the correspondence between T_Linear_ and T_IRT_. Correspondence was especially lacking in the lower range of scores, where T_Linear_ resulted in lower scores compared to T_IRT_ (in 4.88% of the cases the difference was > 5 T-score points; see Table 3). There was more correspondence between T_Rankit_ and T_IRT_, with only 1.5% and 0.78% of the cases differing more than 5 points (see Table 3).

Table 4 presents a cross-walk tables with RS, T_IRT_ scores regressed on RS (CT_IRT_) along with the two PR scores for all possible RS. With nonlinear regression the best fitting curve was found to describe the relation between RS and T_IRT_, yielding the formula for CT_IRT_. Likewise, a curve was found for the relation between RS and PR scores. For these scores, polynomial and sigmoid functions fitted best. These curves could be used to interpolate T_IRT_ or PR_n and PR_cl for RS that do not exist in the dataset. The formulas can also be implemented in software to score questionnaires. The formulas derived from non-linear regression of T or PR onto RS are given below Table 4 in a note.

**Table 4:** Cross-walk Table for the MANSA Total Score

RS	T^1^	PR_n^2^	PR_cl^3^	RS	T^1^	PR_n^2^	PR_cl^3^
12	1.9	0	0	49	36.3	9	44
13	3.9	0	0	50	37.0	10	46
14	5.7	-	0	51	37.8	12	49
15	7.4	-	0	52	38.6	14	51
16	9.0	-	0	53	39.4	16	54
17	10.5	-	1	54	40.2	18	56
18	11.9	0	1	55	41.1	20	59
19	13.2	-	1	56	42.0	23	61
20	14.4	0	1	57	42.9	26	63
21	15.6	-	2	58	43.8	29	65
22	16.7	-	2	59	44.8	33	67
23	17.7	-	2	60	45.8	37	70
24	18.7	0	3	61	46.8	41	72
25	19.6	0	3	62	47.9	45	74
26	20.5	0	4	63	48.9	50	77
27	21.3	0	5	64	50.1	55	79
28	22.1	0	6	65	51.2	60	81
29	22.9	0	6	66	52.4	65	83
30	23.6	0	7	67	53.6	70	85
31	24.4	0	8	68	54.8	75	87
32	25.0	0	10	69	56.1	79	89
33	25.7	1	11	70	57.4	84	90
34	26.4	1	12	71	58.8	88	92
35	27.0	1	13	72	60.2	91	94
36	27.7	1	15	73	61.7	94	95
37	28.3	1	17	74	63.2	96	96
38	28.9	1	19	75	64.7	97	97
39	29.6	2	21	76	66.3	98	98
40	30.2	2	23	77	67.9	99	98
41	30.8	2	25	78	69.6	99	99
42	31.5	3	27	79	71.3	100	99
43	32.1	3	29	80	73.1	100	99
44	32.8	4	31	81	75.0	100	100
45	33.5	5	34	82	76.9	100	100
46	34.1	5	36	83	78.8	100	100
47	34.8	6	39	84	80.9	100	100
48	35.6	8	41				

NB: RS = Raw Score; T = T-score; PR_n = Percentile Rank score general population; PR_cl = Percentile Rank score clinical sample

^1^Formula general population for RS- > T(IRT): y = −3.441e + 01 + 4.302*RS-1.297e-01*RS^2 + 2.152e-03*RS^3–1.705e-05*RS^4 + 5.793e-08*RS^5; a grade 5 polynomial function

^2^Formula general population for RS- > PR_n: y = 4.735e-01 + (1.006e + 02–4.735e-01)*(1-exp(-exp(8.691*(ln(RS +.0001)-ln(6.563e + 01))))); a Weib2 function

^3^Formula clinical sample for RS- > PR_cl: y = −8.841 + 1.916*RS-1.478e-01*RS^2 + 4.878e-03*RS^3–5.504e-05*RS^4 + 2.006e-07*RS^5; a grade 5 polynomial function

**Fig. 5:**
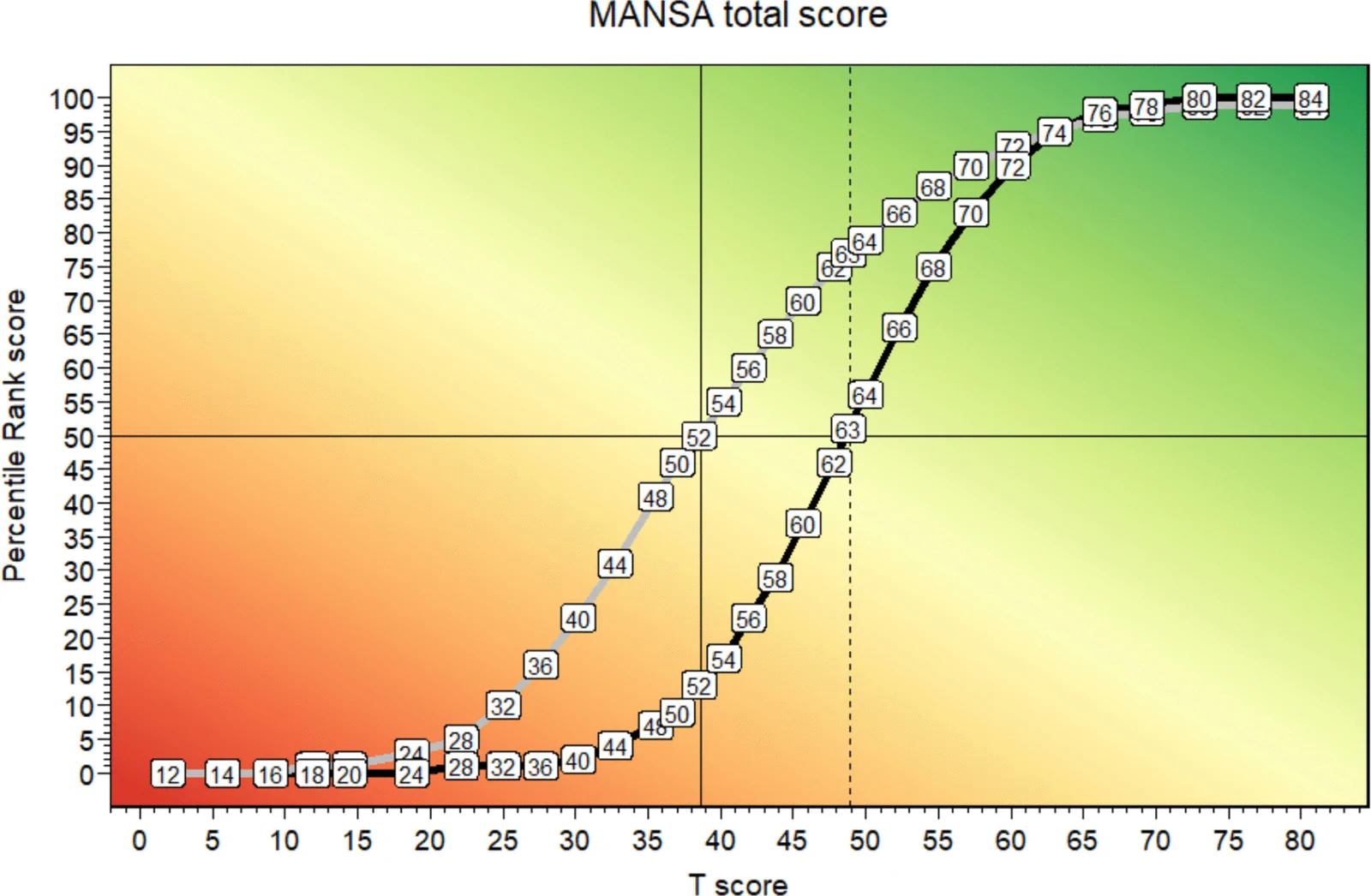
The Relation Between Raw Scores, T-scores, and Two Types of PR-scores. Note: Raw MANSA scores are in the white labels; the line for PR scores for the general population sample (PR_n) is black; for the clinical sample (PR_cl), the line is grey; the solid vertical line is the mean of the clinical sample (RS = 52), the dashed line the mean of the general population sample (RS = 63)

Figure 5 shows for a selection of RS (the even scores) the relation between T-scores and both PR-scores. It illustrates that when the clinical reference group is used, T-scores corresponded to higher PR scores, compared to the general population, reflecting the fact that high MANSA scores were more common in the general population than in the clinical sample. For instance, in the clinical reference group, a raw score of 52 is average and corresponds to a T-score of 38.6. In the general population a raw score of 63 is average and corresponds with a T score of about 50.

### Norms for Gender and Age Groups

**Fig. 6:**
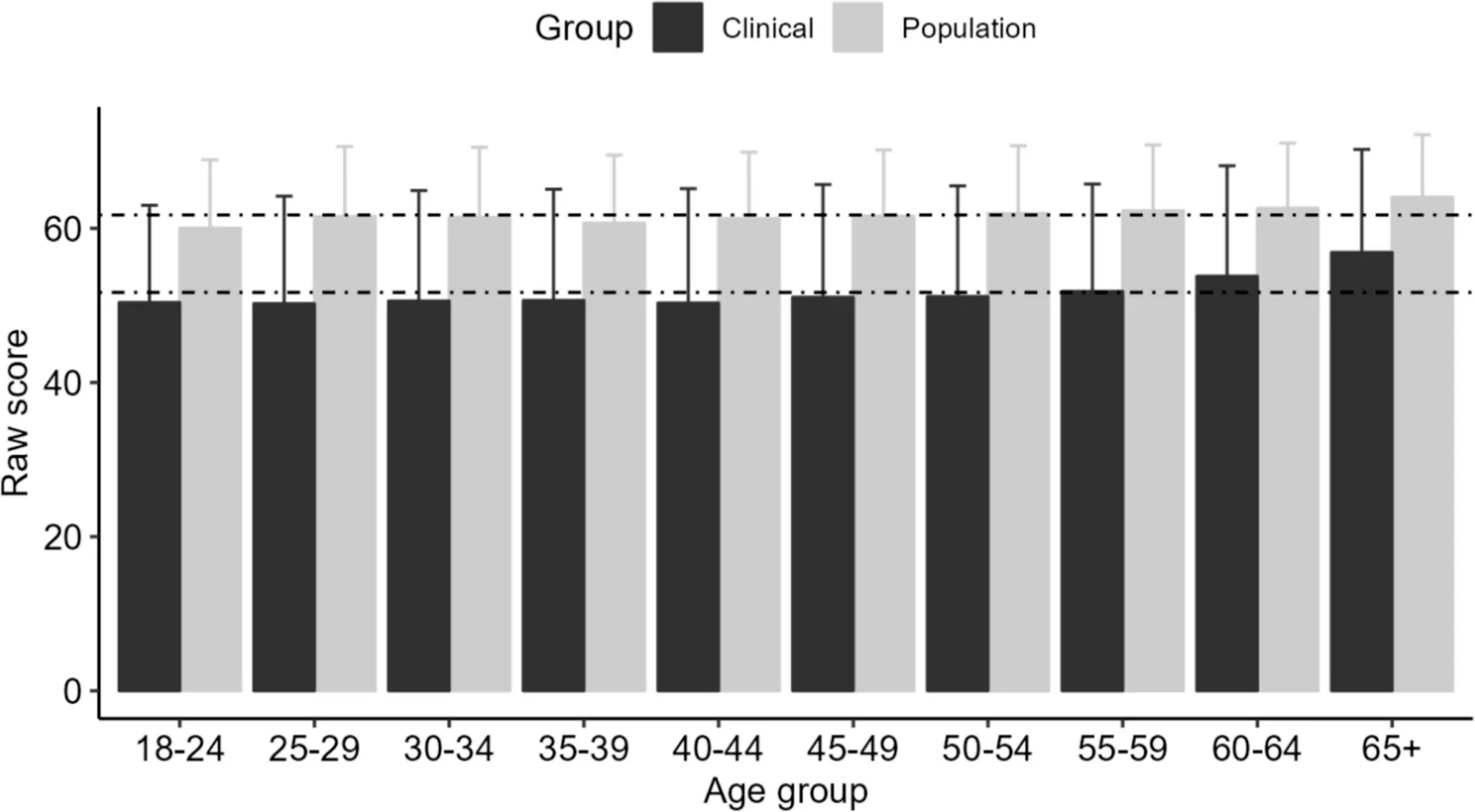
Mean MANSA Scores in the General Population and Clinical Sample for Various Age Groups. Note: Error bars indicate standard deviations; dashed lines are means for the general population (upper line) and clinical subjects (lower line)

Data on the mean (and SD of raw MANSA scores of both samples for various age groups are provided in Table 5. MANSA scores for the various age groups differed because QoL typically rose with age, as shown in Fig. 6.

**Table 5:** Mean scores (and SD) of the General Population and the Clinical Samples for age groups

	General Population			Clinical sample		
		MANSA SCORE			MANSA SCORE	
Age groups	N	M	SD	N	M	SD
18–24	1 304	60.02	8.87	1 058	50.38	12.62
25–29	1 248	61.53	9.08	1 458	50.24	13.94
30–34	964	61.42	9.11	1 400	50.58	14.33
35–39	900	60.69	8.81	1 279	50.65	14.43
40–44	1 070	61.26	8.62	1 091	50.32	14.83
45–49	1 271	61.59	8.59	1 092	51.07	14.62
50–54	1 483	61.93	8.79	897	51.16	14.35
55–59	1 500	62.29	8.54	749	51.82	13.93
60–64	1 128	62.62	8.44	503	53.80	14.32
65 +	921	64.05	8.11	456	56.89	13.35
All	11 789	61.72	8.76	9 983	51.14	14.20

This rise in scores started around age 45. For subsequent analyses and norming of the MANSA, we split the samples on age into two groups (18–44 and 45 +).

MANSA scores of the general population and clinical population and both genders were compared with a two-way Analysis of Variance (ANOVA). A significant interaction effect between gender and clinical status was found which indicated a slightly larger effect of clinical status among woman than men. Five of the six possible differences between subgroups reached significance. As expected, clinical respondents reported a significantly lower QoL than peers from the general population (−10.92 scale points), but further inspection of the means revealed that the gender differences were very small: 0.38 scale points in the general population and 1.11 scale points in the clinical sample.

**Fig. 7:**
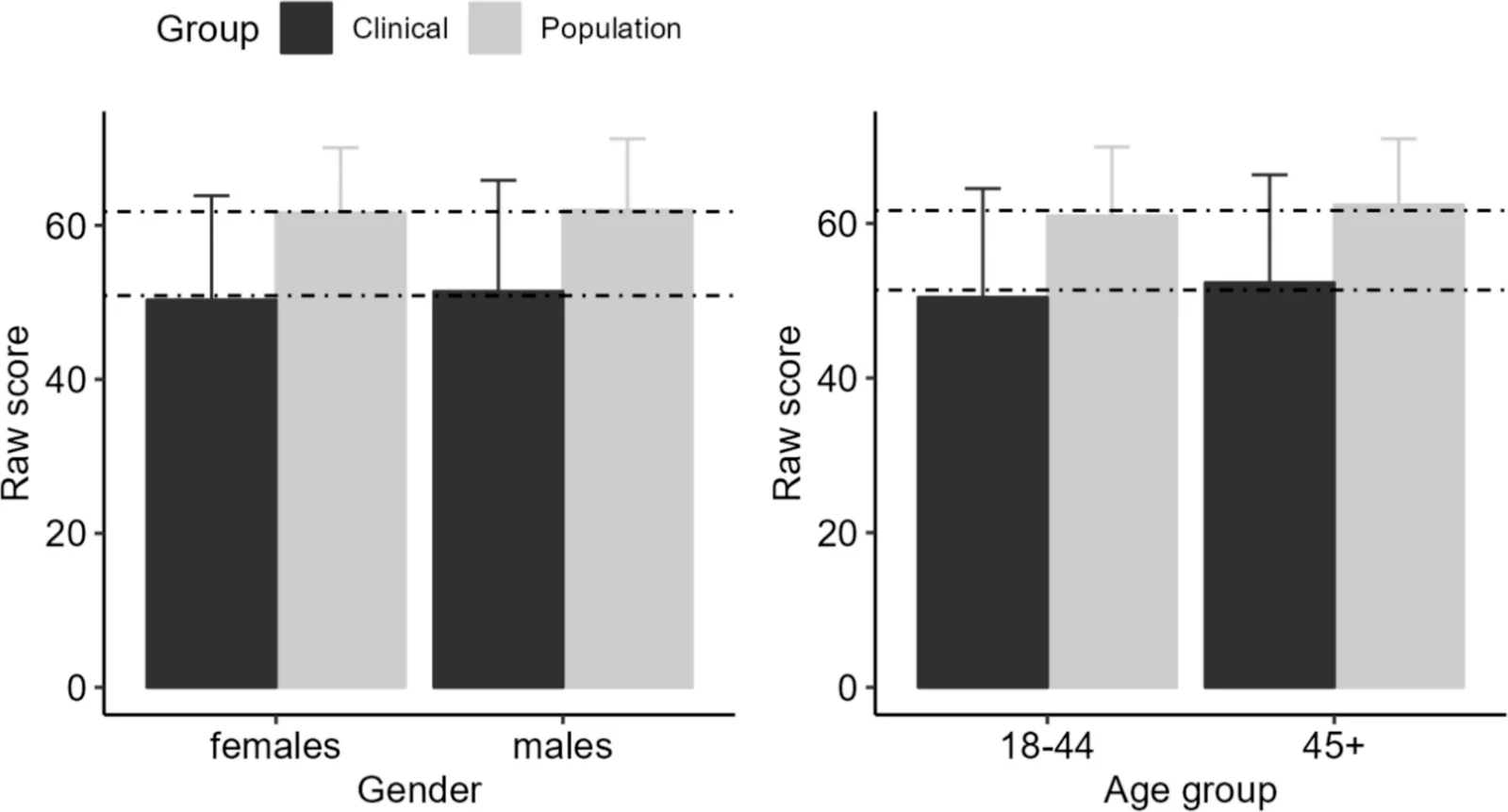
Mean MANSA Scores for clinical status by gender (left) and clinical status by age group (right) with error bars for SD. Note: Dashed lines are means for the general population (upper line) and clinical subjects (lower line)

A similar two-way ANOVA for age group by clinical status (means are presented in Table 5) revealed no interaction effect between both factors, but did reveal main effects as older respondents reported slightly higher QoL (*F*_(1,21768)_ = 104.79; *p* <.001; *Eta*^*2*^ = 0.017), and clinical subjects reported substantially lower QoL (*F*_(1,21768)_ = 4197.07; *p* <.001; *Eta*^*2*^ = 0.172). Figure 3 illustrates the interaction effect for gender and clinical status and the main effects for clinical status and age group. We provide separate cross-walk table for two age groups in the supplementary materials (Fig. 7).

Finally, the general population sample comprised a variable on education level, scored 1 (elementary school not completed) to 8 (academic degree). With each successive point increase in educational level, the reported QoL increased as well (*r* =.18, p <.001). When we split the sample into two groups based on education (low/high), we observed significant lower mean MANSA (QoL) scores among less educated participants (*n* = 7 374; *Mean* = 60.91, *SD* = 9.01) than among higher educated adults (*n* = 4415; *M* = 63.08, *SD* = 8.14), although differences were small (Cohen's *d* = 0.25 [0.21, 0.29]; two-sided *t*_(10037.23)_ = 13.47; *p* <.001) and we did not find it necessary to establish separate norms for education levels. We lacked information on the education level of the clinical subjects, so we did not establish separate norms for them either.

### Reliable and Clinically Significant Change

The data allowed us to compute two indices for the MANSA that were proposed by Jacobson and colleagues (Jacobson & Truax, 1991; Jacobson et al., 1986) to denote clinical relevant outcomes: the Reliable Change Index (RCI) and the cut-off (CO) for recovery. The RCI is an attribute of the instrument, representing the amount of change in score that can be expected based on measurement error of the instrument. A change larger than the RCI-value is likely to represent true change. The CO value is the cut-off value delineating the transition from dysfunctional to functional; a score on the MANSA larger than CO stems more likely from the functional than from the dysfunctional population. Thus, if a change exceeds the RCI, it likely reflects a genuine improvement, while the CO value separates dysfunctional from functional scores on the MANSA, with higher scores indicating a greater likelihood of better functional status. Both combined yield five levels of end-state functioning: Recovered; Reliably Improved, Unchanged; Reliably Deteriorated; Relapsed.

**Fig. 8:**
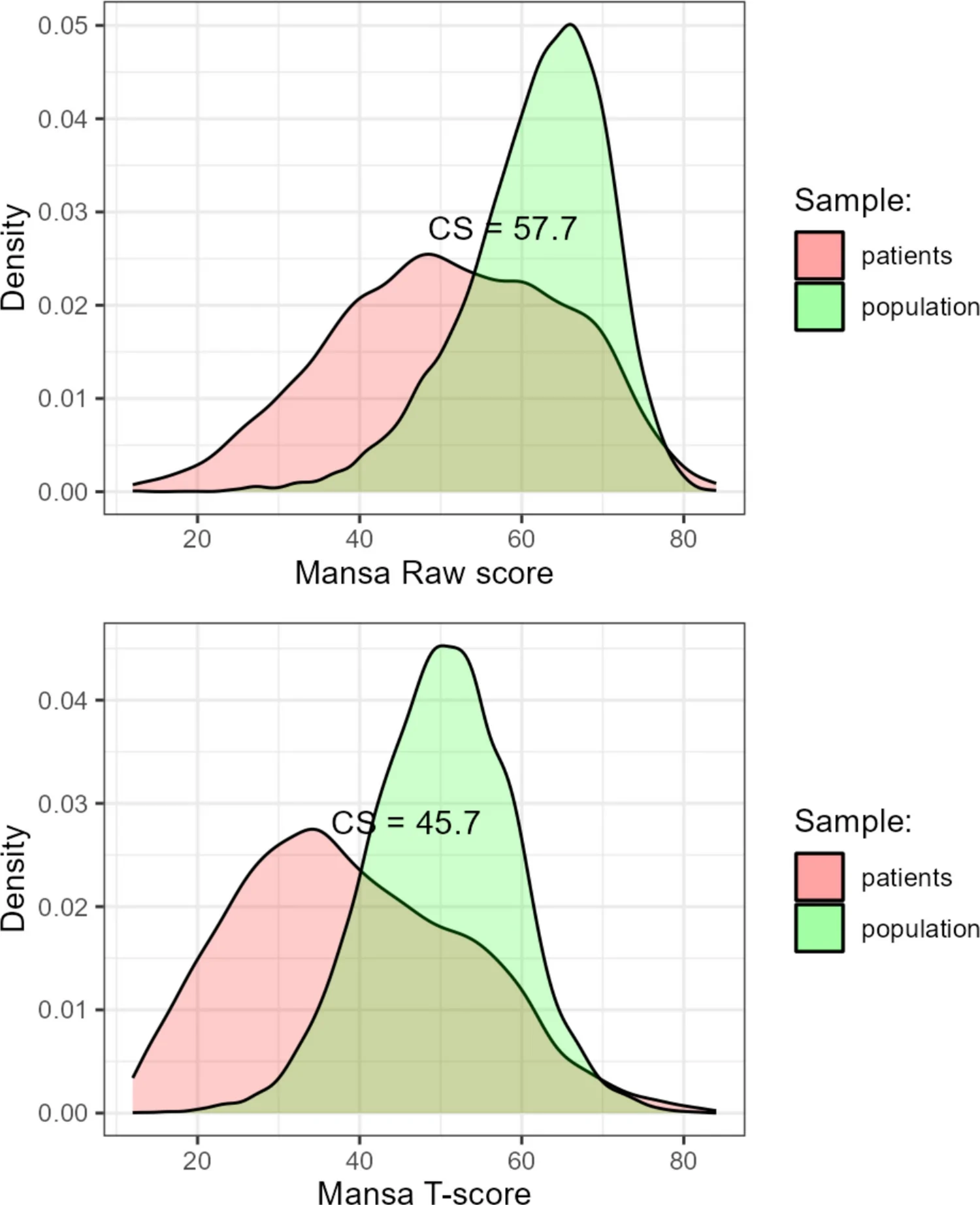
Density plot of the Raw Score Distribution (upper graph) and T-score Distribution (lower graph) of MANSA in the Clinical Sample and the General Population Along With the Clinical Significance cut-off. Note: CS = Cut-off for Clinical Significance

Table 6 presents RCI and CO for raw scores and T-scores of the MANSA and formulas for how these were calculated. Figure 8 shows the CS as the intersection point of the density curves for the general population and the clinical sample for raw scores and for T-scores.

**Table 6:** Reliable Change Index and Clinical Significance for the MANSA Total Score

		RS		T-score
Clinical sample	M	51.14		37.98
(n = 9 983)	SD	14.20		15.69
Normal sample	M	61.72		50.06
(n = 11 789)	SD	8.76		8.86
Reliability	r_xx_ (a)	0.88		0.88
Standard error	S_E_	3.09		3.06
95% RCI	RCI95	8.47		8.57
90% RCI	RCI90	7.11		7.19
80% RCI	RCI80	5.54		5.60
Cut-off	CS	57.68		45.7

NB: RS = raw score; RCI = Reliable Change Index: ${S}_{E}=\mathrm{SD}\sqrt{1-{r}_{xx}}$ and $RCI=1.96*\sqrt{2{{S}_{E}}^{2}}$ and Clinical Significance: $CS= \frac{{sd}_{2}*{M}_{1}+ {sd}_{1}*{M}_{2}}{{sd}_{1}+{sd}_{2}}$

**Fig. 9:**
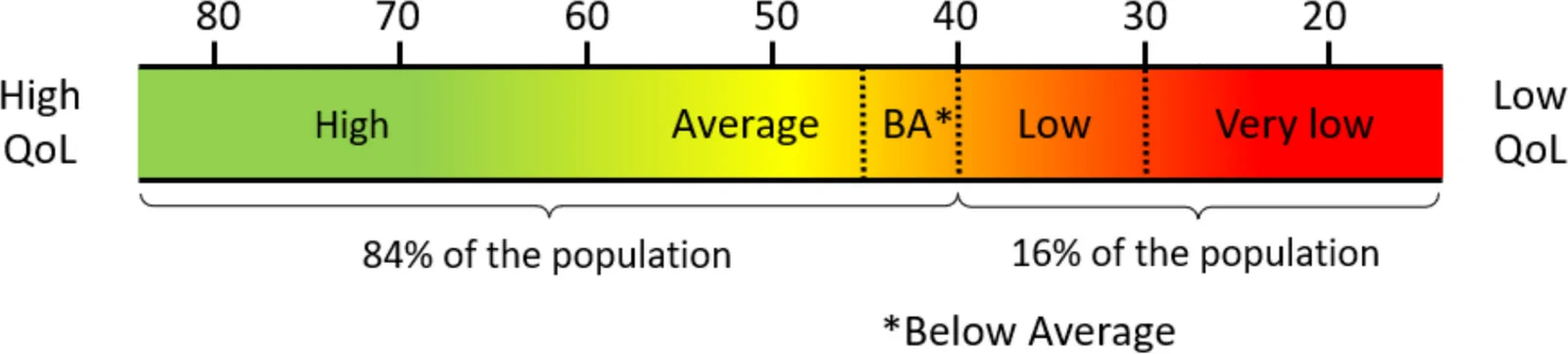
Cut-off Values for Various Levels of Satisfaction with Quality of Life According to T-scores for the MANSA. Note: BA = Below Average

Finally, Fig. 9 shows cut-off scores for various levels in a figure intended to ease interpretation of the T-score, similar to the visualization proposed by the PROMIS initiative (N.B.: See: https://www.healthmeasures.net/score-and-interpret/interpret-scores/promis/promis-score-cut-points).

PROMIS proposed to present T-scores always with decreasing health from left to right, over a heat map from green to red, and higher scores should represent more of the measured construct. This results in a decreasing order of T-score values for constructs as “functioning” or QoL from left to right, as illustrated in Fig. 9.

## Discussion

Key findings of the study were as follows. Raw MANSA total scores from the general population did not have a normal distribution, and revealed an age difference, as satisfaction with QoL rose with age. Consequently, compared to the gold standard of IRT based factor scores (thetas), T-scores established with a linear transformation of raw scores were too low at the lower end of the scale and at the highest extreme. Rankit-based T-scores (T_Rankit_) or T-scores calculated as CT_IRT_ were better approximations of θ-based T-scores. This is demonstrated in Fig. 1 and with Bland–Altman plots for the correspondence of T_Linear_ and T_Rankit_ with T_IRT_ in Fig. 2.

We provided a crosswalk table for the translation of raw scores into T_IRT_ and two types of PR-scores (and provided formulas to calculate all three in scoring software) and a crosswalk figure (Fig. 5), also showing PR-scores, for both samples. Actually, T_Rankit_ and CT_IRT_-scores were so similar, that using the Rankit approach for normalization seems a viable alternative to the more complex IRT-based method, as the latter requires the establishment of an IRT-model on a substantial dataset and stringent assumptions regarding unidimensionality Furthermore, monotonicity of the scale and its items need to be met. The present results align with the pioneering work of Lord and Wingersky (1984) and more recent work of Schalet and colleagues (2021) who found, in the context of test-score equation or linking, that equipercentile linking and IRT-based methods produced highly similar results. The Rankit approach is straightforward and can be done with more traditional statistical software, such as SPSS, SAS, or STATA. To obtain T-scores in clinical practice, the conversion formulas under Table 3 can easily be applied to the raw score of an individual patient (e.g., with MS-Excel), in order to obtain T- and PR scores. An excel file with the formulas is included in the supplementary materials.

Furthermore, the finding of an age difference necessitates age-appropriate norms and T- and PR scores, when the aim is to interpret scores of individual patients. Tables with age-adjusted T- and PR scores are provided in the supplementary materials. However, if the research question pertains to differences in MANSA scores between age groups, non-adjusted T- and PR scores should be used, as age-adjusted scores will be on average 50 for both age groups.

Finally, clinical significance cut-off values for RCI and CS were established for raw scores and T_IRT_-scores. For the latter, an RCI95 of at least8.5 T-score points was found, a stringent criterion, as this comes close to requiring almost a standard deviation of change. A test user could also consider a more lenient RCI90, which requires at least 7.2 change in T-score. A cut-off score for Clinical Significance (the transition of dysfunctional to functional) was determined at 45.7, which coincides with the transition of “Average” to “Below Average” for T-scores propagated by the PROMIS initiative (see Fig. 9).

Using tests, such as the MANSA, to evaluate satisfaction with QoL of our clients at the start of treatment and to monitor progress over time is an essential element of evidence-based care (Lewis et al., 2019) and routine monitoring of outcomes is included in treatment guidelines (Prevolnik Rupel et al., 2021). Measurement is also recommended by researchers warning for the unreliability of unstructured clinical judgment (Kahneman et al., 2021). Despite the established benefits (Delgadillo et al., 2017; Lambert & Harmon, 2018), standardized assessment remains underutilized (Boswell et al., 2013; Jensen-Doss et al., 2018). One of the reasons for hesitation in the field may be difficulty with interpretation of test results. We hope that our earlier plea (de Beurs et al., 2022) to make use of standard measurement scales, such as T- and PR-scores, will stimulate standardized assessment in the therapy office.

### Strengths and Limitations

A strength of this research is sizable samples to fit an IRT model and to establish norms for two age groups (18–44 and 45 +). There was some overrepresentation of women in the general population sample and underrepresentation in the clinical sample of patients with substance use disorder. However, no gender difference was found in MANSA scores of both samples and adjustment of T- and PR scores for gender did not appear necessary. Some unresearched issues remain, such as, investigation of other background or demographic variables, that may affect the test score, e.g., socioeconomic status or highest attained education level. As we used anonymized patient data, we had no access to this information for the clinical sample and could not investigate influence of these factors. Transforming raw scale score to T-scores will make it easier to understand the test result, as a score below 50 will mean sub-average satisfaction with QoL, whereas 16% of the population scores below 40 and 16% above 60.

## Conclusion

Utilizing common metrics, such as T-scores and PR-scores, when reviewing test results with patients, can facilitate their understanding of the meaning test results. This approach may bridge a gap between the psychometric expertise of the professional and the everyday experience of the client. It may also facilitate discussions about the implications of the test results for tailoring therapeutic interventions and may help to determine when therapeutic goals have been achieved and treatment can be concluded.

## Supplementary Information

Below is the link to the electronic supplementary material.

Supplementary file1 (XLSX 18 KB)

Supplementary file2 (DOCX 130 KB)
