# Supplementary material for: The Devil Is in the Tail: Manchester Short Assessment of Quality of Life (MANSA) test percentiles and normalized T-scores
Source: J Psychopathol Behav Assess. 2026 Jan 28;48(1):5. doi: 10.1007/s10862-025-10266-0 (PMC12847227; doi:10.1007/s10862-025-10266-0)
Supplement: Supplementary file 2 — Supplementary file2 (DOCX 130 KB) [file 10862_2025_10266_MOESM2_ESM.docx]

Table S-1. Cross-walk Table for the MANSA Total Score for two age groups.

|  | 18-44 | | | 45+ | | |
| --- | --- | --- | --- | --- | --- | --- |
| RS | T1^1^ | PR_n1^2^ | PR_cl1^3^ | T2^4^ | PR_n2^5^ | PR_cl2^6^ |
| 12 | -7.4 | 0 | 0 | -8.1 | - | 0 |
| 13 | -5.4 | - | 0 | -5.9 | 0 | 0 |
| 14 | -3.4 | - | 0 | -3.9 | - | 0 |
| 15 | -1.6 | - | 0 | -2.0 | - | 0 |
| 16 | 0.1 | - | 0 | -0.2 | - | 0 |
| 17 | 1.7 | - | 0 | 1.5 | - | 0 |
| 18 | 3.2 | - | 0 | 3.0 | 0 | 0 |
| 19 | 4.6 | - | 1 | 4.5 | - | 0 |
| 20 | 6.0 | 0 | 1 | 5.9 | - | 0 |
| 21 | 7.3 | - | 1 | 7.2 | - | 1 |
| 22 | 8.6 | - | 2 | 8.5 | - | 1 |
| 23 | 9.8 | - | 2 | 9.7 | - | 2 |
| 24 | 11.0 | 0 | 3 | 10.8 | 0 | 2 |
| 25 | 12.1 | 0 | 4 | 12.0 | - | 3 |
| 26 | 13.2 | 0 | 4 | 13.0 | 0 | 3 |
| 27 | 14.3 | 0 | 5 | 14.1 | 0 | 4 |
| 28 | 15.4 | 0 | 6 | 15.1 | 0 | 5 |
| 29 | 16.5 | 0 | 7 | 16.1 | 0 | 6 |
| 30 | 17.5 | 0 | 8 | 17.1 | 0 | 7 |
| 31 | 18.6 | 0 | 9 | 18.1 | 0 | 8 |
| 32 | 19.6 | 0 | 11 | 19.1 | 0 | 9 |
| 33 | 20.7 | 0 | 12 | 20.0 | 0 | 10 |
| 34 | 21.7 | 0 | 13 | 21.0 | 0 | 11 |
| 35 | 22.7 | 0 | 15 | 22.0 | 0 | 13 |
| 36 | 23.7 | 0 | 17 | 22.9 | 0 | 14 |
| 37 | 24.8 | 1 | 18 | 23.9 | 0 | 15 |
| 38 | 25.8 | 1 | 20 | 24.8 | 0 | 17 |
| 39 | 26.8 | 2 | 22 | 25.8 | 1 | 19 |
| 40 | 27.8 | 2 | 24 | 26.7 | 1 | 20 |
| 41 | 28.8 | 3 | 26 | 27.7 | 2 | 22 |
| 42 | 29.9 | 3 | 29 | 28.7 | 2 | 24 |
| 43 | 30.9 | 4 | 31 | 29.6 | 3 | 26 |
| 44 | 31.9 | 5 | 33 | 30.6 | 3 | 28 |
| 45 | 32.9 | 6 | 36 | 31.6 | 4 | 30 |
| 46 | 33.9 | 7 | 38 | 32.5 | 5 | 33 |
| 47 | 35.0 | 8 | 40 | 33.5 | 6 | 35 |
| 48 | 36.0 | 9 | 43 | 34.5 | 7 | 37 |
| 49 | 37.0 | 11 | 46 | 35.4 | 8 | 39 |
| 50 | 38.0 | 12 | 48 | 36.4 | 9 | 42 |
| 51 | 39.0 | 14 | 51 | 37.4 | 11 | 44 |
| 52 | 40.0 | 16 | 53 | 38.4 | 12 | 47 |
| 53 | 41.0 | 18 | 56 | 39.3 | 14 | 49 |
| 54 | 42.0 | 20 | 58 | 40.3 | 16 | 52 |
| 55 | 43.0 | 23 | 61 | 41.3 | 18 | 54 |
| 56 | 44.0 | 26 | 63 | 42.3 | 21 | 57 |
| 57 | 45.0 | 29 | 66 | 43.2 | 23 | 59 |
| 58 | 46.0 | 32 | 68 | 44.2 | 26 | 62 |
| 59 | 47.1 | 36 | 70 | 45.2 | 30 | 64 |
| 60 | 48.1 | 40 | 73 | 46.2 | 34 | 67 |
| 61 | 49.1 | 44 | 75 | 47.3 | 38 | 69 |
| 62 | 50.2 | 49 | 77 | 48.3 | 42 | 71 |
| 63 | 51.2 | 54 | 79 | 49.4 | 47 | 74 |
| 64 | 52.3 | 59 | 81 | 50.4 | 52 | 76 |
| 65 | 53.4 | 64 | 83 | 51.5 | 57 | 78 |
| 66 | 54.6 | 69 | 85 | 52.7 | 62 | 80 |
| 67 | 55.7 | 73 | 86 | 53.8 | 68 | 82 |
| 68 | 57.0 | 78 | 88 | 55.1 | 73 | 84 |
| 69 | 58.2 | 82 | 89 | 56.3 | 78 | 86 |
| 70 | 59.6 | 86 | 91 | 57.6 | 82 | 88 |
| 71 | 61.0 | 89 | 92 | 59.0 | 86 | 89 |
| 72 | 62.4 | 92 | 93 | 60.5 | 89 | 91 |
| 73 | 64.0 | 94 | 95 | 62.0 | 92 | 93 |
| 74 | 65.7 | 96 | 96 | 63.6 | 95 | 94 |
| 75 | 67.4 | 98 | 97 | 65.4 | 96 | 95 |
| 76 | 69.3 | 99 | 98 | 67.2 | 98 | 96 |
| 77 | 71.3 | 100 | 99 | 69.2 | 99 | 98 |
| 78 | 73.4 | 100 | 100 | 71.3 | 100 | 99 |
| 79 | 75.8 | 100 | 100 | 73.5 | 100 | 100 |
| 80 | 78.2 | 100 | 100 | 76.0 | 100 | 100 |
| 81 | 80.9 | 100 | 100 | 78.6 | 100 | 100 |
| 82 | 83.8 | - | 100 | 81.4 | 100 | 100 |
| 83 | 86.9 | 100 | 100 | 84.5 | 100 | 100 |
| 84 | 90.3 | 100 | 100 | 87.7 | 100 | 100 |

NB: RS = Raw Score; T = T-score; PR_n = Percentile Rank score general population; PR_cl = Percentile Rank score clinical sample.

^1^Formula for normal 18-44 (ID:3) for RS->TIRT: y=-47.88+5.102*RS-0.1898*RS^2+0.004377*RS^3-0.00004995*RS^4+0.0000002251*RS^5; a poly5 function.

^2^Formula for normal 18-44 (ID:13) for RS->PR_n: y=-2.7+105.837/((1+exp(-2.714e-01*(x-67.847)))^4.052e-01); a sigm-L5 function.

^3^Formula for clinical 18-44 (ID:18) for RS->PR_cl: y=109.1-110.202/((1+(x/75.685)^3.891)^3.255); a logis5 function.

^4^Formula for normal 45+ (ID:3) for RS->TIRT: y=-52.92+5.714*RS-0.2158*RS^2+0.004831*RS^3-0.00005345*RS^4+0.0000002346*RS^5; a poly5 function.

^5^Formula for normal 45+ (ID:13) for RS->PR_n: y=-2.5+105.265/((1+exp(-2.975e-01*(x-69.108)))^3.858e-01); a sigm-L5 function.

^6^Formula for clinical 45+ (ID:17) for RS->PR_cl: y=18-44 jr; a Weib2 function.

Figure S-1.

Screen shot of Excel file for scoring MANSA


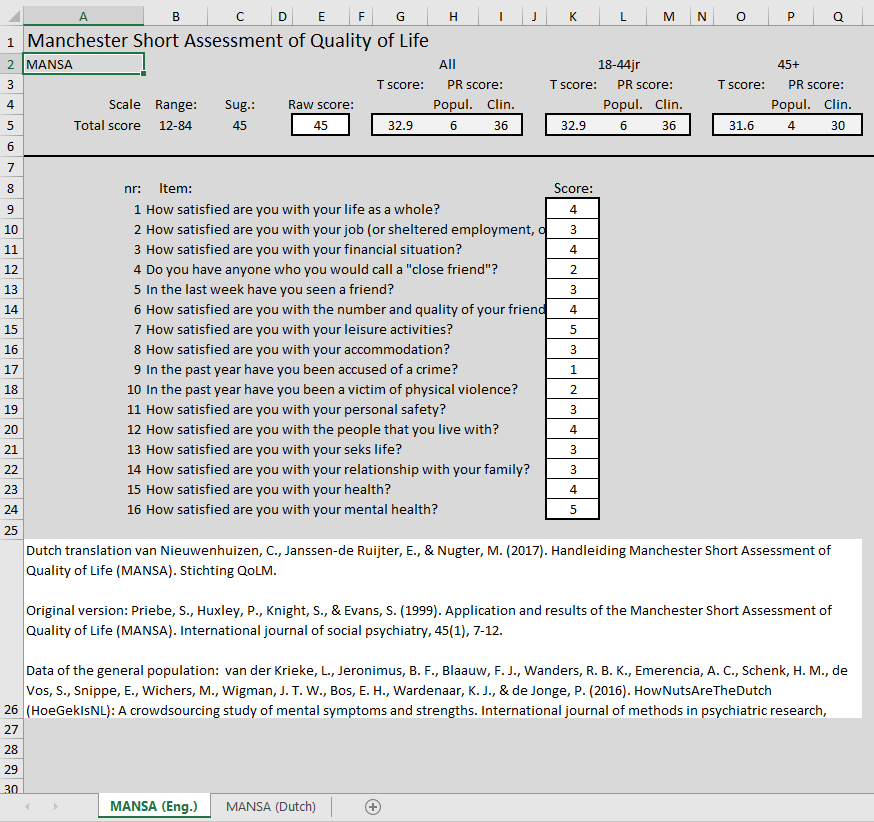


Use:

On this worksheet, the raw score can be entered under "Raw score:". The worksheet then immediately calculates T- and PR scores.

The worksheet also allows for the entry of a respondent's item scores. The Excel worksheet then first calculates the scale score(s) and adds them to the column under "Sug:" for suggested score. The user can enter the suggested scale score into the "Raw score:" field. The Excel worksheet then immediately calculates normalized T-scores and PR scores for each raw score.
